# Supplementary material for: Early-life intestinal microbiome in Trachemys scripta elegans analyzed using 16S rRNA sequencing
Source: PeerJ. 2020 Feb 5;8:e8501. doi: 10.7717/peerj.8501 (PMC7007735; doi:10.7717/peerj.8501)
Supplement: Supplemental Information 1 [file peerj-08-8501-s001.docx]

**Supplementary figures and tables**

**
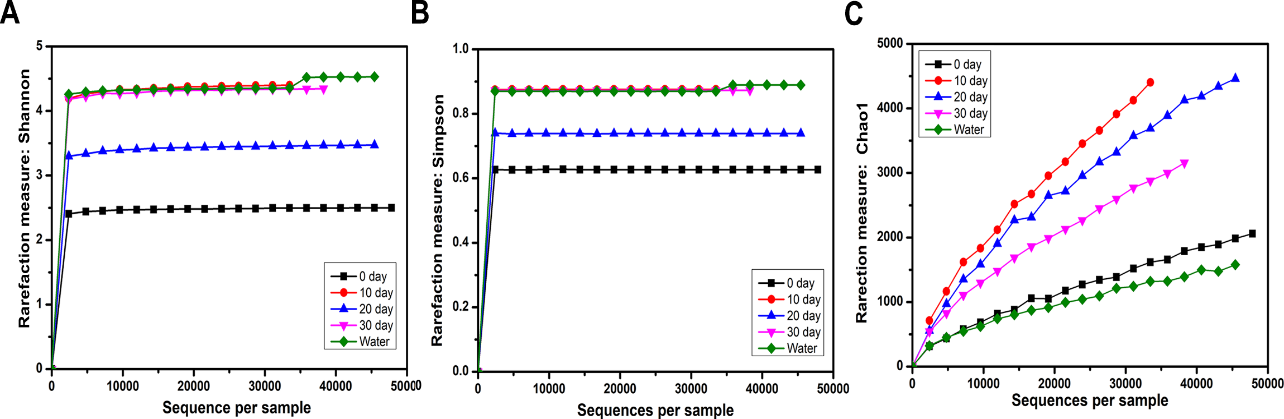
**

**Figure S1. Alpha diversity analysis of microbiomes from turtle intestines at different growth stages and of the environmental water.** (A) the Shannon index, (B) the Simpson index, (C) the Chao1 estimator.

**Table S1. The distribution of dominant bacteria at the phylum level in turtle intestine at different growth stages and in the environmental water.**

| Dominate phylum | Mean of relative abundance (%) | | | | |
| --- | --- | --- | --- | --- | --- |
|  | 0-day | 10-day | 20-day | 30-day | Water |
| Actinobacteria | 0.03 | 1.06 | 0.00 | 0.29 | 4.45 |
| Bacteroidetes | 0.32 | 0.31 | 27.17 | 22.55 | 23.24 |
| Firmicutes | 58.23 | 92.94 | 67.08 | 56.46 | 1.90 |
| Proteobacteria | 41.42 | 5.68 | 5.74 | 20.66 | 70.33 |
| Cyanobacteria | 0 | 0 | 0 | 0 | 0.02 |

**Table S2. The distribution of dominant bacteria at the family level in turtle intestine at different growth stages and in the environmental water.**

| Dominate Family | Mean of OTUs (%) | | | | |
| --- | --- | --- | --- | --- | --- |
|  | 0-day | 10-day | 20-day | 30-day | Water |
| Weeksellaceae | 0.12 | 0.21 | 0.01 | 0.04 | 8.95 |
| Aeromonadaceae | 0.00 | 0.00 | 0.05 | 0.10 | 12.18 |
| Alcaligenaceae | 10.76 | 0.11 | 0.00 | 0.00 | 1.09 |
| Bacteroidaceae | 0.01 | 0.01 | 27.36 | 22.56 | 0.26 |
| Clostridiaceae | 13.03 | 30.24 | 9.39 | 18.13 | 0.16 |
| Comamonadaceae | 0.01 | 0.02 | 0.02 | 0.13 | 22.86 |
| Cytophagaceae | 0.00 | 0.00 | 0.00 | 0.00 | 7.71 |
| Enterobacteriaceae | 23.52 | 0.40 | 5.34 | 12.56 | 4.56 |
| Lachnospiraceae | 0.74 | 33.75 | 27.74 | 8.18 | 0.14 |
| Methylobacteriaceae | 0.73 | 0.13 | 0.04 | 2.85 | 3.63 |
| Moraxellaceae | 0.02 | 3.75 | 0.01 | 2.30 | 7.78 |
| Paenibacillaceae | 36.01 | 0.86 | 0.00 | 0.00 | 0.04 |
| Peptostreptococcaceae | 0.57 | 19.94 | 23.43 | 17.60 | 0.07 |
| Ruminococcaceae | 0.00 | 0.00 | 3.00 | 3.64 | 0.06 |
| Staphylococcaceae | 0.01 | 4.07 | 0.00 | 0.02 | 0.43 |
| Xanthomonadaceae | 0.37 | 1.27 | 0.01 | 0.03 | 7.84 |

**Table S3. The distribution of dominant bacteria at the genus level at different growth stages of turtle intestine and in the environmental water.**

| Dominate Genus | Mean of OTUs (%) | | | | |
| --- | --- | --- | --- | --- | --- |
|  | 0-day | 10-day | 20-day | 30-day | Water |
| *Achromobacter* | 10.75 | 0.10 | 0.00 | 0.00 | 1.05 |
| *Acinetobacter* | 0.02 | 3.75 | 0.01 | 2.29 | 7.69 |
| *Citrobacter* | 20.78 | 0.24 | 5.17 | 11.77 | 3.40 |
| *Cloacibacterium* | 0.00 | 0.00 | 0.00 | 0.02 | 6.94 |
| *Clostridium* | 11.95 | 24.98 | 8.23 | 17.50 | 0.15 |
| *Comamonas* | 0.01 | 0.00 | 0.01 | 0.08 | 17.95 |
| *Epulopiscium* | 0.15 | 31.91 | 1.42 | 0.72 | 0.02 |
| *Flectobacillus* | 0.00 | 0.00 | 0.00 | 0.00 | 6.66 |
| *Methylobacterium* | 0.73 | 0.13 | 0.04 | 2.85 | 3.60 |
| *Paenibacillus* | 36.01 | 0.86 | 0.00 | 0.00 | 0.04 |
| *Stenotrophomonas* | 0.37 | 1.21 | 0.00 | 0.01 | 6.79 |

**Table S4. The core OTUs existing in all the turtle intestinal samples**

| OTU ID | Taxonomy |
| --- | --- |
| OTU15730 | k__Bacteria; p__Firmicutes; c__Clostridia; o__Clostridiales; f__Clostridiaceae; g__; s__ |
| OTU9929 | k__Bacteria; p__Firmicutes; c__Clostridia; o__Clostridiales; f__Clostridiaceae; g__; s__ |
| OTU20347 | k__Bacteria; p__Firmicutes; c__Clostridia; o__Clostridiales; f__Peptostreptococcaceae; g__; s__ |
| OTU23770 | k__Bacteria; p__Firmicutes; c__Clostridia; o__Clostridiales; f__Clostridiaceae; g__SMB53; s__ |
| OTU16404 | k__Bacteria; p__Firmicutes; c__Clostridia; o__Clostridiales; f__Clostridiaceae; g__SMB53; s__ |
| OTU4788 | k__Bacteria; p__Firmicutes; c__Clostridia; o__Clostridiales; f__Peptostreptococcaceae; g__; s__ |
| OTU15234 | k__Bacteria; p__Firmicutes; c__Clostridia; o__Clostridiales; f__Peptostreptococcaceae; g__; s__ |
| OTU15090 | k__Bacteria; p__Firmicutes; c__Clostridia; o__Clostridiales; f__Peptostreptococcaceae; g__; s__ |
| OTU14103 | k__Bacteria; p__Firmicutes; c__Clostridia; o__Clostridiales; f__Clostridiaceae; g__; s__ |
| OTU8288 | k__Bacteria; p__Firmicutes; c__Clostridia; o__Clostridiales; f__Peptostreptococcaceae; g__; s__ |
| OTU13236 | k__Bacteria; p__Firmicutes; c__Clostridia; o__Clostridiales; f__Peptostreptococcaceae; g__; s__ |
| OTU5062 | k__Bacteria; p__Firmicutes; c__Clostridia; o__Clostridiales; f__Peptostreptococcaceae; g__; s__ |
| OTU23626 | k__Bacteria; p__Firmicutes; c__Clostridia; o__Clostridiales; f__Lachnospiraceae; g__; s__ |
| OTU21165 | k__Bacteria; p__Firmicutes; c__Clostridia; o__Clostridiales; f__Clostridiaceae; g__Clostridium; s__perfringens |
| OTU15627 | k__Bacteria; p__Firmicutes; c__Clostridia; o__Clostridiales; f__Clostridiaceae; g__Clostridium; s__perfringens |
| OTU565 | k__Bacteria; p__Firmicutes; c__Clostridia; o__Clostridiales; f__Clostridiaceae; g__; s__ |
| OTU12087 | k__Bacteria; p__Firmicutes; c__Clostridia; o__Clostridiales; f__Clostridiaceae; g__SMB53; s__ |
| OTU110 | k__Bacteria; p__Firmicutes; c__Clostridia; o__Clostridiales; f__Peptostreptococcaceae; g__; s__ |
| OTU22190 | k__Bacteria; p__Firmicutes; c__Clostridia; o__Clostridiales; f__Lachnospiraceae; g__Epulopiscium; s__ |
| OTU204 | k__Bacteria; p__Firmicutes; c__Clostridia; o__Clostridiales; f__Clostridiaceae; g__; s__ |
| OTU7060 | k__Bacteria; p__Firmicutes; c__Clostridia; o__Clostridiales; f__Clostridiaceae; g__; s__ |
| OTU12011 | k__Bacteria; p__Firmicutes; c__Clostridia; o__Clostridiales; f__Clostridiaceae; g__; s__ |
| OTU15421 | k__Bacteria; p__Firmicutes; c__Clostridia; o__Clostridiales; f__Clostridiaceae; g__Clostridium; s__perfringens |
| OTU11755 | k__Bacteria; p__Firmicutes; c__Clostridia; o__Clostridiales; f__Clostridiaceae; g__Sarcina; s__ |
| OTU4315 | k__Bacteria; p__Firmicutes; c__Clostridia; o__Clostridiales; f__Clostridiaceae; g__Clostridium; s__perfringens |
| OTU10548 | k__Bacteria; p__Firmicutes; c__Clostridia; o__Clostridiales; f__Clostridiaceae; g__Sarcina; s__ |
| OTU16273 | k__Bacteria; p__Firmicutes; c__Clostridia; o__Clostridiales; f__Clostridiaceae; g__Clostridium; s__perfringens |
| OTU17309 | k__Bacteria; p__Firmicutes; c__Clostridia; o__Clostridiales; f__Clostridiaceae; g__; s__ |
| OTU4166 | k__Bacteria; p__Firmicutes; c__Clostridia; o__Clostridiales; f__Clostridiaceae; g__Clostridium; s__perfringens |
| OTU8421 | k__Bacteria; p__Firmicutes; c__Clostridia; o__Clostridiales; f__Clostridiaceae; g__SMB53; s__ |
| OTU11871 | k__Bacteria; p__Firmicutes; c__Clostridia; o__Clostridiales; f__Clostridiaceae; g__; s__ |
| OTU11506 | k__Bacteria; p__Firmicutes; c__Clostridia; o__Clostridiales; f__Peptostreptococcaceae; g__; s__ |
| OTU13936 | k__Bacteria; p__Firmicutes; c__Clostridia; o__Clostridiales; f__Lachnospiraceae; g__Epulopiscium; s__ |
| OTU13309 | k__Bacteria; p__Firmicutes; c__Clostridia; o__Clostridiales; f__Clostridiaceae; g__SMB53; s__ |
| OTU3969 | k__Bacteria; p__Firmicutes; c__Clostridia; o__Clostridiales; f__Clostridiaceae; g__; s__ |
| OTU6686 | k__Bacteria; p__Firmicutes; c__Clostridia; o__Clostridiales; f__Clostridiaceae |
| OTU23839 | k__Bacteria; p__Firmicutes; c__Clostridia; o__Clostridiales; f__Clostridiaceae; g__; s__ |
| OTU15338 | k__Bacteria; p__Firmicutes; c__Clostridia; o__Clostridiales; f__Clostridiaceae; g__; s__ |
| OTU12421 | k__Bacteria; p__Firmicutes; c__Clostridia; o__Clostridiales; f__Clostridiaceae |
| OTU15827 | k__Bacteria; p__Firmicutes; c__Clostridia; o__Clostridiales; f__Clostridiaceae; g__; s__ |
| OTU946 | k__Bacteria; p__Firmicutes; c__Clostridia; o__Clostridiales; f__Lachnospiraceae; g__Epulopiscium; s__ |
| OTU212 | k__Bacteria; p__Firmicutes; c__Clostridia; o__Clostridiales; f__Clostridiaceae; g__SMB53; s__ |
| OTU19626 | k__Bacteria; p__Firmicutes; c__Clostridia; o__Clostridiales; f__Clostridiaceae; g__; s__ |
| OTU21976 | k__Bacteria; p__Firmicutes; c__Clostridia; o__Clostridiales; f__Peptostreptococcaceae; g__; s__ |
| OTU23987 | k__Bacteria; p__Firmicutes; c__Clostridia; o__Clostridiales; f__Peptostreptococcaceae; g__; s__ |
| OTU12220 | k__Bacteria; p__Firmicutes; c__Clostridia; o__Clostridiales; f__Clostridiaceae; g__Clostridium; s__perfringens |
| OTU14227 | k__Bacteria; p__Firmicutes; c__Clostridia; o__Clostridiales; f__Clostridiaceae; g__Clostridium; s__perfringens |

**Table S5. LEfSe analysis of all the intestinal samples from turtles**

| Groups | Taxonomy | Highest Mean | LDA SCORE (log 10) | P-Value |
| --- | --- | --- | --- | --- |
| 0-day | Bacteria.Firmicutes.Bacilli.Bacillales.Paenibacillaceae | 5.556 | 5.265 | 0.017 |
|  | Bacteria.Firmicutes.Bacilli | 5.568 | 5.257 | 0.029 |
|  | Bacteria.Firmicutes.Bacilli.Bacillales.Paenibacillaceae.Paenibacillus | 5.541 | 5.247 | 0.017 |
|  | Bacteria.Firmicutes.Bacilli.Bacillales.Paenibacillaceae.Paenibacillus.lautus | 5.567 | 5.244 | 0.014 |
|  | Bacteria.Firmicutes.Bacilli.Bacillales | 5.504 | 5.233 | 0.030 |
|  | Bacteria.Proteobacteria.Betaproteobacteria.Burkholderiales.Alcaligenaceae | 4.562 | 4.642 | 0.042 |
|  | Bacteria.Proteobacteria.Betaproteobacteria.Burkholderiales.Alcaligenaceae.Achromobacter | 4.376 | 4.626 | 0.035 |
|  | Bacteria.Proteobacteria.Alphaproteobacteria.Rhizobiales.Brucellaceae.Ochrobactrum | 2.432 | 3.879 | 0.048 |
|  | Bacteria.Firmicutes.Bacilli.Bacillales.Bacillaceae.Bacillus | 3.700 | 3.749 | 0.028 |
|  | Bacteria.Firmicutes.Bacilli.Bacillales.Bacillaceae.Bacillus.firmus | 2.991 | 3.433 | 0.042 |
|  | Bacteria.Bacteroidetes.Sphingobacteriia.Sphingobacteriales.Sphingobacteriaceae.Sphingobacterium | 3.793 | 2.994 | 0.028 |
|  | Bacteria.Bacteroidetes.Sphingobacteriia.Sphingobacteriales | 3.700 | 2.975 | 0.023 |
|  | Bacteria.Bacteroidetes.Sphingobacteriia.Sphingobacteriales.Sphingobacteriaceae | 3.377 | 2.972 | 0.028 |
|  | Bacteria.Bacteroidetes.Sphingobacteriia | 3.442 | 2.919 | 0.023 |
| 10-day | Bacteria.Firmicutes.Clostridia.Clostridiales | 5.934 | 5.515 | 0.050 |
|  | Bacteria.Firmicutes.Clostridia | 5.934 | 5.499 | 0.050 |
|  | Bacteria.Firmicutes.Clostridia.Clostridiales.Lachnospiraceae.Epulopiscium | 5.528 | 5.209 | 0.027 |
|  | Bacteria.Firmicutes.Clostridia.Clostridiales.Lachnospiraceae | 5.447 | 5.195 | 0.050 |
|  | Bacteria.Actinobacteria.Actinobacteria.Actinomycetales.Intrasporangiaceae.Janibacter | 3.120 | 3.500 | 0.042 |
|  | Bacteria.Actinobacteria.Actinobacteria.Actinomycetales.Brevibacteriaceae | 5.447 | 3.288 | 0.042 |
|  | Bacteria.Actinobacteria.Actinobacteria.Actinomycetales.Brevibacteriaceae.Brevibacterium | 5.031 | 3.263 | 0.042 |
|  | Bacteria.Actinobacteria.Actinobacteria.Actinomycetales.Brevibacteriaceae.Brevibacterium.aureum | 4.710 | 3.263 | 0.042 |
|  | Bacteria.Firmicutes.Bacilli.Bacillales.Planococcaceae.Kurthia.gibsonii | 3.793 | 3.005 | 0.013 |
|  | Bacteria.Firmicutes.Bacilli.Bacillales.Planococcaceae | 3.626 | 2.988 | 0.025 |
|  | Bacteria.Firmicutes.Bacilli.Bacillales.Planococcaceae.Kurthia | 3.045 | 2.954 | 0.013 |
| 20-day | Bacteria.Bacteroidetes | 5.437 | 5.153 | 0.040 |
|  | Bacteria.Bacteroidetes.Bacteroidia | 5.436 | 5.152 | 0.038 |
|  | Bacteria.Bacteroidetes.Bacteroidia.Bacteroidales.Bacteroidaceae | 5.032 | 5.148 | 0.038 |
|  | Bacteria.Bacteroidetes.Bacteroidia.Bacteroidales | 4.777 | 5.128 | 0.038 |
|  | Bacteria.Firmicutes.Clostridia.Clostridiales.Clostridiaceae.Clostridium.butyricum | 3.844 | 3.907 | 0.041 |
|  | Bacteria.Firmicutes.Clostridia.Clostridiales.Lachnospiraceae.Coprococcus | 3.045 | 3.606 | 0.032 |
|  | Bacteria.Firmicutes.Clostridia.Clostridiales.Lachnospiraceae.Dorea | 2.031 | 3.523 | 0.028 |
|  | Bacteria.Firmicutes.Bacilli.Turicibacterales.Turicibacteraceae.Turicibacter | 3.176 | 3.522 | 0.034 |
|  | Bacteria.Firmicutes.Bacilli.Turicibacterales | 2.653 | 3.504 | 0.034 |
|  | Bacteria.Firmicutes.Bacilli.Turicibacterales.Turicibacteraceae | 3.040 | 3.468 | 0.034 |
|  | Bacteria.Bacteroidetes.Bacteroidia.Bacteroidales.Odoribacteraceae.Odoribacter | 3.599 | 2.959 | 0.013 |
|  | Bacteria.Firmicutes.Clostridia.Clostridiales.Ruminococcaceae.Faecalibacterium | 1.888 | 2.881 | 0.040 |
|  | Bacteria.Bacteroidetes.Bacteroidia.Bacteroidales.Odoribacteraceae | 2.031 | 2.877 | 0.013 |
|  | Bacteria.Firmicutes.Erysipelotrichi.Erysipelotrichales.Erysipelotrichaceae.Eubacterium | 2.975 | 2.731 | 0.022 |
|  | Bacteria.Firmicutes.Erysipelotrichi.Erysipelotrichales.Erysipelotrichaceae.Eubacterium.dolichum | 2.975 | 2.721 | 0.022 |
| 30-day | Bacteria.Firmicutes.Clostridia.Clostridiales.Lachnospiraceae.Blautia | 4.211 | 4.077 | 0.017 |
|  | Bacteria.Firmicutes.Erysipelotrichi.Erysipelotrichales | 4.101 | 4.049 | 0.014 |
|  | Bacteria.Firmicutes.Erysipelotrichi.Erysipelotrichales.Erysipelotrichaceae | 4.101 | 4.044 | 0.014 |
|  | Bacteria.Firmicutes.Erysipelotrichi | 3.956 | 4.025 | 0.014 |
|  | Bacteria.Firmicutes.Clostridia.Clostridiales.Ruminococcaceae.Ruminococcus | 3.626 | 3.808 | 0.042 |
|  | Bacteria.Bacteroidetes.Cytophagia.Cytophagales | 1.888 | 3.685 | 0.024 |
|  | Bacteria.Bacteroidetes.Cytophagia | 3.377 | 3.677 | 0.024 |
|  | Bacteria.Bacteroidetes.Cytophagia.Cytophagales.Cytophagaceae | 3.599 | 3.657 | 0.024 |
|  | Bacteria.Tenericutes | 3.376 | 3.532 | 0.013 |
|  | Bacteria.Tenericutes.Mollicutes.RF39 | 3.109 | 3.461 | 0.013 |
|  | Bacteria.Firmicutes.Clostridia.Clostridiales.Ruminococcaceae.Anaerotruncus | 2.982 | 3.442 | 0.013 |
|  | Bacteria.Proteobacteria.Alphaproteobacteria.Rhizobiales.Phyllobacteriaceae.Chelativorans | 2.493 | 3.372 | 0.040 |
|  | Bacteria.Bacteroidetes.Bacteroidia.Bacteroidales.Porphyromonadaceae | 4.317 | 3.208 | 0.040 |
|  | Bacteria.Tenericutes.Mollicutes | 4.317 | 3.173 | 0.013 |
|  | Bacteria.Firmicutes.Clostridia.Clostridiales.Veillonellaceae | 4.233 | 3.167 | 0.017 |
|  | Bacteria.Firmicutes.Clostridia.Clostridiales.Lachnospiraceae.Blautia.producta | 1.697 | 3.133 | 0.024 |
|  | Bacteria.Firmicutes.Clostridia.Clostridiales.Peptococcaceae | 2.432 | 2.922 | 0.025 |
|  | Bacteria.Firmicutes.Erysipelotrichi.Erysipelotrichales.Erysipelotrichaceae.Holdemania | 2.975 | 2.752 | 0.013 |
|  | Bacteria.Actinobacteria.Actinobacteria.Actinomycetales.Microbacteriaceae.Clavibacter | 2.659 | 2.640 | 0.013 |
|  | Bacteria.Firmicutes.Clostridia.Clostridiales.Lachnospiraceae.Ruminococcus | 2.493 | 2.611 | 0.040 |
|  | Bacteria.Firmicutes.Clostridia.Clostridiales.Lachnospiraceae.Ruminococcus.gnavus | 2.686 | 2.605 | 0.040 |

**
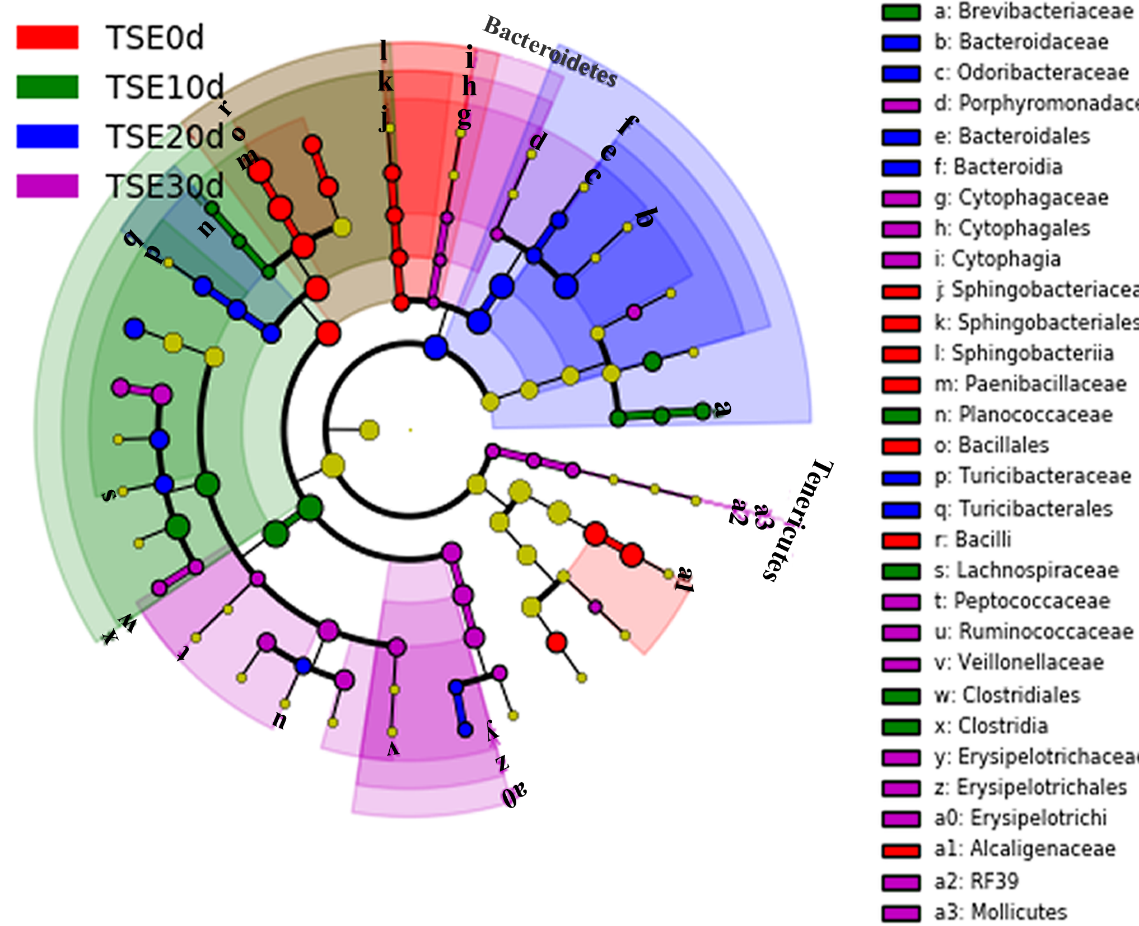
**

**Figure S2.** **Taxonomic cladogram obtained from LEfSe analysis of 16S rRNA sequences (relative abundance ≥ 0.5%).** The enriched taxa of TSE0d (Red), TSE10d (Green), TSE20d (Blue), and TSE30d (Purple) were shown. 0d, 10d, 20d, 30d indicate the intestine microbiome of turtles for growing 0 day, 10days, 20 days and 30 days. Only taxa meeting an LDA significant threshold >2 are shown.

**Table S6. List of DNA sequence used in this study**

| Sequence number | Group | Sample ID |
| --- | --- | --- |
| D1700911 | 0-day | TSE0d1 |
| D1700912 |  | TSE0d2 |
| D1700913 |  | TSE0d3 |
| D1700917 | 10-day | TSE10d1 |
| D1700918 |  | TSE10d2 |
| D1700919 |  | TSE10d3 |
| D1700923 | 20-day | TSE20d1 |
| D1700924 |  | TSE20d2 |
| D1700925 |  | TSE20d3 |
| D1700935 | 30-day | TSE30d1 |
| D1700936 |  | TSE30d2 |
| D1700937 |  | TSE30d3 |
| D1700947 | Water | TSEe1 |
| D1700950 |  | TSEe2 |
| D1700953 |  | TSEe3 |
